# Why stable dietary guidelines produce different meanings: an interpretive framework for early guideline impact

**DOI:** 10.3389/fnut.2026.1845445

**Published:** 2026-06-16

**Authors:** Abeer Almudaihim

**Affiliations:** 1 Department of Clinical Nutrition, College of Applied Medical Sciences, King Saud bin Abdulaziz University for Health Sciences, Riyadh, Saudi Arabia; 2 King Abdullah International Medical Research Center, Riyadh, Saudi Arabia; 3 Department of Clinical Nutrition, Ministry of National Guard - Health Affairs, Riyadh, Saudi Arabia

**Keywords:** dietary guidelines, framing and salience, guideline interpretation, implementation science, nutrition education, policy translation, professional practice, public health policy

## Abstract

Dietary guidelines are usually assessed through downstream behavioral or health outcomes, often treating early interpretation as a neutral step in dissemination. Yet guidelines function as broader communication packages that include framing, food-based emphasis, visual presentation, and simplified public messaging, all of which may shape how guidance is understood before behavioral changes become measurable. The 2025–2030 Dietary Guidelines for Americans illustrate this process: increased public emphasis on protein, full-fat dairy, and animal fats may influence how guidance is interpreted and communicated, even when structural continuity in long-standing numerical recommendations remains. Drawing on implementation science, I propose the Early Interpretive Pathway of Dietary Guideline Impact (EIP-DGI), a conceptual framework that examines guideline impact across three stages: early public interpretation (Phase I), educational and professional translation (Phase II), and downstream behavioral outcomes (Phase III). Unlike frameworks primarily focused on organizational adoption, EIP-DGI identifies the period immediately following guideline release as an early and assessable stage of impact. This perspective has implications for how guideline dissemination and uptake are evaluated across educational, clinical, and cultural settings.

## 1 Introduction

Evaluation frameworks for dietary guidelines have traditionally focused on numerical targets and the scientific evidence supporting them, with impact usually assessed through dietary behavior or health outcomes (1, 2). Retained quantitative thresholds may contribute to perceptions of continuity across guideline cycles (3–5). As a result, less attention is often given to how guidance is interpreted during dissemination, with interpretation more commonly discussed later as part of implementation or behavioral response (6, 7). This can make early shifts in meaning harder to identify as guidelines move into public and professional use. In practice, dietary guidance is often communicated through framing, visual presentation, food-based emphasis, and simplified public messaging rather than through numerical recommendations alone (8, 9). Greater visibility of certain foods or dietary themes in consumer-facing materials may therefore shape how guidance is understood during early dissemination. This interpretive limitation becomes visible in the 2025–2030 Dietary Guidelines for Americans (DGA). Although the DGA retained the long-standing recommendation to limit saturated fat to less than 10% of total daily energy, early public and professional discourse has placed greater emphasis on protein intake, full-fat dairy, and animal-based foods (3, 10, 11). This creates an important interpretive issue: formal numerical continuity does not necessarily ensure continuity in how guidance is understood, prioritized, or translated into practice. Implementation science frameworks have developed concepts and methods for studying guideline uptake across settings. The Consolidated Framework for Implementation Research (CFIR) in its original and updated forms (12, 13) and the Knowledge-to-Action (KTA) framework (14, 15) acknowledge that uptake occurs within complex contextual systems (6, 16, 17). These frameworks have been applied largely in healthcare and clinician-mediated adoption contexts (18–20), where interpretation is generally treated as part of broader implementation processes rather than as a phase of impact. As a result, early differences in how guidelines are understood and translated into practice may remain difficult to identify within these frameworks.

This perspective presents the Early Interpretive Pathway of Dietary Guideline Impact (EIP-DGI), a conceptual framework based on implementation science to examine early interpretive processes following the publication of dietary guidelines, using the 2025–2030 DGA as an illustrative example. The framework was developed through the integration of implementation science theory and analysis of recent dietary guideline discourse. EIP-DGI proposes that interpretation itself represents an early and assessable stage of guideline impact and that some variation in uptake may originate during dissemination before implementation differences or behavioral outcomes become visible. I also propose that interpretive pathways vary across cultural and institutional contexts, where the same numerical recommendations may attach to different food categories and produce different practical meanings across settings (4, 21–24).

## 2 The EIP-DGI framework

### 2.1 Core definitions

Structural continuity refers to the persistence of formal numerical recommendations or threshold-based guidance from one cycle to the next. Retained numerical thresholds may also function as reference points when guideline cycles are compared during evaluation. Interpretive variability refers to differences in how guidance is prioritized, interpreted, and perceived during dissemination, even when the formal recommendations remain the same, drawing on framing theory’s distinction between message content and salience (25). Early interpretation refers to the short period after guideline release during which practical meaning is built through media discourse, institutional communication, educational translation, and professional simplification (8, 16).

### 2.2 Three phases of dietary guideline impact

**Figure 1:**
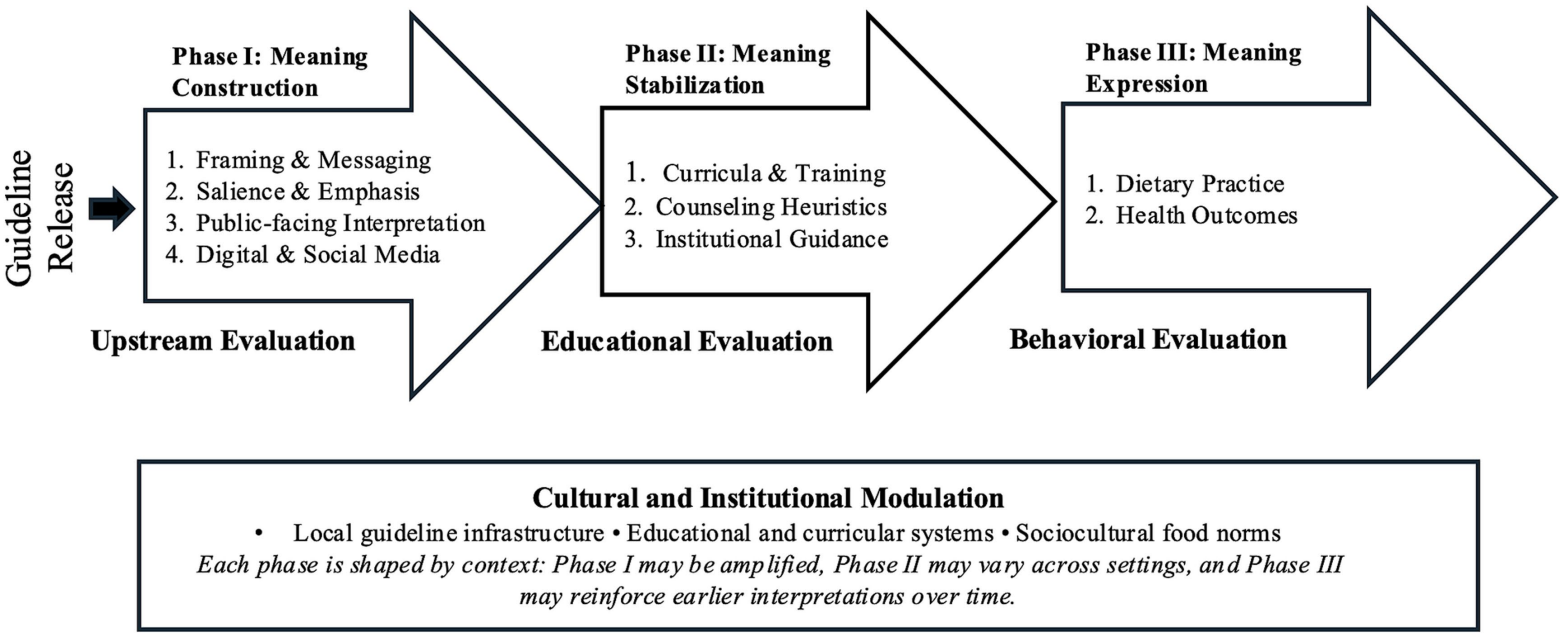
The early interpretive pathway of dietary guideline impact (EIP-DGI). This presents three sequential phases of guideline influence: early interpretation during dissemination (phase I—meaning construction), educational and institutional stabilization (phase II—meaning stabilization), and downstream dietary practice and health outcomes (phase III—meaning expression). Each phase is associated with a corresponding evaluation domain: upstream evaluation, educational evaluation, and behavioral evaluation. The lower contextual layer indicates that interpretation and uptake may be shaped by cultural and institutional factors, including local guideline infrastructure, educational systems, and sociocultural food norms.

EIP-DGI proposes three analytically distinct phases linked in sequence (Figure 1). Phase I—Meaning Construction covers the earliest stages of dissemination, when guideline meaning is built through public-facing communication. Phase II—Meaning Stabilization is where the early interpretive signals from Phase I settle and are integrated into educational, clinical, and institutional settings. Phase III—Meaning Expression refers to the downstream behavioral and population-level signals following long-term exposure to the earlier processes.

#### 2.2.1 Phase I: meaning construction—public-facing interpretation and media amplification

Interpretation of dietary guidelines begins with exposure through public-facing channels rather than through formal education or clinical settings (8, 16, 26). EIP-DGI positions the dissemination of consumer-facing summaries through traditional media, institutional communications, and digital platforms as the first stage of the guidelines’ influence. Evidence from dissemination and implementation science suggests that these early interpretive frames influence how guidelines are initially understood and prioritized, often before technical reconciliation has occurred (7, 8, 16, 27).

This is especially true in the 2025–2030 DGA cycle, during which consumer-facing emphasis on prioritizing protein, “real food,” and the inclusion of full-fat dairy has received disproportionate attention in media and professional discourse (3, 28, 29). The 2025–2030 cycle introduced an inverted-pyramid-style visual accompanied by the “Eat Real Food” framework, with greater emphasis on protein-rich foods, full-fat dairy, and animal-based fats such as butter and beef tallow relative to earlier DGA imagery (3, 28, 29). Earlier DGA editions instead focused on dietary patterns built around vegetables, whole grains, and low-fat dairy within the “Make Every Bite Count” framework (3, 11). In public-facing summaries, visually emphasized foods and high-salience take-home messages may receive more attention than the broader dietary context or numerical limits elsewhere in the guidance. Consequently, early public interpretation may reflect the messages that are most visible and repeatedly circulated rather than the full balance of the guideline itself (26, 27).

Professional societies responding to the 2025–2030 DGA have raised some concerns about confusion, inconsistent interpretation, and difficulties in practical application (30–32). Several academic commentaries have highlighted tensions between recommendations on protein intake and on saturated fat and have suggested that simplified public messaging could lead to incomplete or misleading interpretations of the guidance (29–34). As a result, public understanding may begin forming before educational and clinical guidance is fully established. When highly visible messaging dominates the early release period, these interpretations may be reinforced before educators and clinicians have the opportunity to provide clarification, thereby increasing the burden on later Phase II educational and institutional translation efforts.

#### 2.2.2 Phase II: meaning stabilization—educational and professional translation

Phase II is the mediating stage in which interpretations from Phase I begin to settle into curricula, counseling heuristics, professional standards, and continuing education content (7, 9). Educational and professional translation may reinforce some interpretations more strongly during dissemination (35–37). Recommendations that are consistently included in training materials, exams, and institutional guidance tend to have a more lasting influence over time (38–41). Some forms of guidelines may also be easier to communicate in counseling settings than recommendations that require more explanation or context (36, 38, 42). Curricular revision, accreditation standards, professional guidance, and continuing education can all influence which messages become more prominent in practice (38, 40, 41). The influence of Phase I on downstream behavior may depend on the efficiency of Phase II translation (16, 19, 20). In settings with active professional guidance and timely curriculum adjustment, early interpretive variability may be moderated during dissemination (38–41). In contrast, when local guideline infrastructure or educational adaptation is limited, Phase I messaging may have greater downstream influence. In the 2025–2030 cycle, educators and professional groups must interpret consumer-facing messaging alongside existing dietary guidance (3). Because educational and professional materials often adapt more slowly than media coverage and public discussion (16, 39–41), early messaging may shape public understanding before formal guidance and training have fully adjusted.

#### 2.2.3 Phase III: meaning expression—behavioral and population-level outcomes

In Phase III, behavioral and population-level outcomes are shaped by earlier interpretive and educational processes that influence how dietary guidance is understood in practice. Within EIP-DGI, differences in behavior are not interpreted only as non-adherence (35). Traditional views of non-adherence generally assume that people understand the guideline as intended but do not consistently follow it in practice. EIP-DGI instead treats behavioral differences as potentially reflecting divergent interpretations of the guideline, rather than failure to follow a recommendation that everyone understood the same way. Distinguishing between non-adherence and interpretive variability requires measuring interpretation alongside behavior through surveys of clinicians, educators, and the public, together with interrupted time-series analyses following guideline release (26, 43). Changes in public discussion shortly after guideline release may therefore reflect evolving interpretation rather than evidence of guideline effectiveness (6, 9, 26).

#### 2.2.4 Cultural modulation of interpretive pathways: applying EIP-DGI beyond United States contexts

Cross-cultural dissemination is where interpretive modulation becomes most visible. The 2025–2030 DGA was designed for the United States population, but its influence reaches into settings where national dietary guidance is limited or weakly implemented (4, 44), and Phase I circulates widely before Phase II adaptation can occur. In many Arab dietary contexts, meat, full-fat dairy, and traditional animal fats hold culturally important roles within food practices, hospitality, and norms of provision (22, 23). Emphasis on these foods, together with “real food” messaging in the DGA, may contribute to perceptions of endorsement and shifts in dietary interpretation within local food cultures (8, 27, 29, 33). Regional dietary guidelines and pictorial food guides in Arabic-speaking countries often use culturally familiar food groupings, symbolic representations, and locally adapted dietary messaging (44–46). Externally circulating dietary messaging does not enter a neutral interpretive space but interacts with existing cultural meanings attached to food categories and dietary practices (8, 45, 46). Interpretive variability may become more pronounced in settings where traditional dietary patterns remain centered around olive oil, plant fats, whole grains, legumes, and low-fat dairy products, or where regional dietary recommendations continue to emphasize these foods (24, 36, 45, 46). In such settings, clinicians and educators may encounter competing interpretive frameworks in which highly visible international messaging leads some patients to perceive earlier recommendations regarding saturated fat, low-fat dairy, or traditional Mediterranean dietary patterns as outdated, even while structural continuity in the underlying numerical limits remains. The influence of competing interpretations may also depend on which sources of dietary guidance are viewed as more credible or authoritative within local clinical and public settings (37). In some Arab settings, Phase I messaging may play a larger role in shaping interpretation before professional clarification becomes available (16, 17, 44). This suggests that the relative influence of Phase I and Phase II may vary across settings depending on local institutional capacity and access to professional guidance.

## 3 Discussion

Interpretation is not only a downstream consequence of guideline dissemination; it also shapes how guidelines begin to function in practice. Framing, message salience, and educational translation can influence how guidance is understood before behavioral changes become visible (8, 25). Existing implementation science frameworks recognize that uptake occurs across multiple stages, but evaluation still tends to focus more heavily on behavioral outcomes than on earlier interpretive processes (12–15).

Because early interpretation develops through different dissemination mechanisms and timelines than later phases, it may require different forms of evaluation. Phase I can be examined through discourse analysis of media coverage and content analysis of consumer-facing communication materials (8, 25, 27). Phase II may be examined through analyses of educational curricula, professional society statements, counseling protocols, and continuing education resources (21, 38–41). Phase III involves assessing population dietary intake trends using interrupted time-series analyses to examine whether trajectories shift following guideline release (43).

A reasonable objection is that the early interpretive processes described in EIP-DGI may already be captured by existing implementation science frameworks, particularly CFIR and KTA. CFIR’s inner- and outer-setting constructs describe organizational determinants of implementation, while KTA focuses on stages of knowledge translation into practice (12–15). However, both frameworks are anchored mainly in organizational uptake and implementation processes rather than in the early public release period. Within KTA or CFIR, divergence would mainly be interpreted as emerging during implementation or knowledge translation. EIP-DGI instead proposes that some divergence may begin earlier, during dissemination itself, before educators and clinicians enter implementation settings, and therefore treats the pre-implementation interpretive period as a distinct phase of guideline impact. If this earlier origin is not accounted for, variation in uptake may be interpreted primarily as implementation failure or non-adherence, and interventions targeting implementation may be applied to a problem that began upstream.

EIP-DGI is theoretically closest to framing theory, which distinguishes frames embedded in messages from frames constructed through individual interpretation and identifies salience and emphasis as important influences on interpretation (8, 25). However, framing theory primarily explains how messages shape meaning, whereas EIP-DGI focuses on when these interpretive processes emerge within the guideline dissemination pathway and why distinguishing them from later implementation processes matters analytically. In EIP-DGI, early interpretation is examined as an upstream stage of guideline impact rather than as a background feature of communication alone.

EIP-DGI yields three testable predictions. When Phase I foregrounds food-based themes without clarifying retained numerical thresholds despite structural continuity, Phase II educational materials should show measurable divergence before the next guideline cycle (16, 38–41). In settings where local dietary guidelines are absent or weakly implemented, Phase I messaging may play a larger role and later educational translation may show greater interpretive divergence (16, 21, 37). Applied specifically to the 2025–2030 DGA cycle, EIP-DGI predicts that Phase II curricular and professional educational materials may show divergence around the retained ≤10% saturated fat threshold during the early dissemination period, including variability in how the retained limit is emphasized relative to protein-focused and full-fat dairy messaging before Phase III behavioral changes become detectable. This prediction could be examined through content analysis of curricular materials, professional society guidance, and continuing education resources from 2026 onward.

Unequal access to interpretation and clarification may also shape how guidelines are understood in practice. Individuals with limited health literacy, limited access to registered dietitians, or greater reliance on general media may move through Phase I without later educational or professional clarification reaching them, leaving early public messaging as the main version of the guideline they rely on in practice (16, 21, 42). Under these conditions, some population differences in dietary behavior may be interpreted too quickly as non-adherence even when groups are working from different understandings of the guideline. This may be especially relevant in cross-national settings where Phase I messaging circulates globally while educational and professional systems vary substantially between settings (21, 37).

## 4 Conclusion

The early impact of dietary guidelines is best understood as an interpretive process that precedes later behavioral outcomes. Using the 2025–2030 DGA as an example, EIP-DGI shows how shifts in framing and dissemination can influence understanding even when some quantitative recommendations remain stable. Early reactions, media discourse, and professional debate should therefore not be interpreted too quickly as evidence of effectiveness or failure. Evaluation may instead benefit from incorporating early interpretive indicators alongside downstream behavioral measures.

## Data Availability

The original contributions presented in the study are included in the article/supplementary material, further inquiries can be directed to the corresponding author.
